# A Metagenomic Study Highlights Phylogenetic Proximity of Quorum-Quenching and Xenobiotic-Degrading Amidases of the AS-Family

**DOI:** 10.1371/journal.pone.0065473

**Published:** 2013-06-07

**Authors:** Mélanie Tannières, Amélie Beury-Cirou, Armelle Vigouroux, Samuel Mondy, Franck Pellissier, Yves Dessaux, Denis Faure

**Affiliations:** 1 Institut des Sciences du Végétal, UPR2355, Centre National de la Recherche Scientifique, Gif-sur-Yvette, France; 2 Comité Nord Plants de Pommes de Terre (CNPPT), Semences, Innovation, Protection, Recherche et Environnement (SIPRE), Achicourt, France; 3 Laboratoire d’Enzymologie et Biochimie Structurales (LEBS), UPR3082, Centre National de la Recherche Scientifique, Gif-sur-Yvette, France; 4 Institut de Chimie des Substances Naturelles, UPR2301, Centre National de la Recherche Scientifique, Gif-sur-Yvette, France; Université Paris Sud, France

## Abstract

Quorum-sensing (QS) signals of the N-acylhomoserine lactone (NAHL) class are cleaved by quorum-quenching enzymes, collectively named NAHLases. Here, functional metagenomics allowed the discovery of a novel bacterial NAHLase in a rhizosphere that was treated with γ-caprolactone. As revealed by *rrs*-DGGE and *rrs*-pyrosequencing, this treatment increased the percentage of the NAHL-degrading bacteria and strongly biased the structure of the bacterial community, among which *Azospirillum* dominated. Among the 29 760 fosmids of the metagenomic library, a single one was detected that expressed the *qsdB* gene conferring NAHL-degradation upon *E. coli* and decreased QS-regulated virulence in *Pectobacterium*. Phylogenetic analysis of the 34 *orfs* of the fosmid suggested that it would belong to an unknown Proteobacterium - probably a γ-proteobacterium. qPCR quantification of the NAHLase-encoding genes *attM, qsdA, and qsdB* revealed their higher abundance in the γ-caprolactone-treated rhizosphere as compared to an untreated control. The purified QsdB enzyme exhibited amidase activity. QsdB is the first amidase signature (AS) family member exhibiting NAHLase-activity. Point mutations in the AS-family catalytic triad K-S-S abolished the NAHLase activity of QsdB. This study extends the diversity of NAHLases and highlights a common phylogenic origin of AS-family enzymes involved in the degradation of natural compounds, such as NAHLs, and xenobiotics, such as nylon and linuron.

## Introduction

N-acylhomoserine lactones (NAHLs) are diffusible signals used by many *Proteobacteria* to correlate gene expression to cell density via a regulatory pathway called quorum-sensing (QS) [1]–[2]. Aside from the enzymes implicated in biosynthesis of NAHLs, others are able to cleave or modify NAHLs, hence to disrupt QS-signaling [3]. They have been identified in bacteria and eukaryotes, and are collectively called NAHLases or quorum-quenching enzymes [4]. According to their structures and enzymatic activities, NAHLases are classified in different families: the lactonases of the Zn-hydrolase family, such as AiiA, AttM/BlcC, AhlD, and QlcA [5]–[9]; the lactonases of the alpha/beta hydrolase fold family such as AidH and AiiM [10]–[11]; the lactonases of the phosphotriesterase family with a single NAHLase member, QsdA [12]; the amidases of the amidohydrolase cluster of the beta-lactam acylases, such as AiiD, PvdQ, AhlM, AiiC, and QuiP [13]–[17]; and the short-chain reductase family with one NAHLase member, the reductase BpiB09 [18]–[19]. The biological role(s) of NAHLases generally remains unclear, except in some firmicutes where they may contribute to the degradation of toxic NAHL-derivatives [20], or in the plant pathogen *Agrobacterium tumefaciens* in which they slightly modulate QS-functions [21]–[22]. In spite of this lack of information on the biological roles of NAHLases, these enzymes have been used successfully to quench QS-regulated functions. For instance, virulence in the plant pathogen *Pectobacterium* could be reduced or abolished either via the production of transgenic plants expressing NAHLase-encoding gene [23] or by the selection of bacterial isolates or populations exhibiting NAHLase activities [24]–[27].

Functional metagenomics, which includes screening of environmental-DNA libraries for enzymatic activities or metabolite synthesis, emerged as a powerful approach to explore and exploit the natural biodiversity of microorganisms [28]–[30]. In the investigated metagenomic libraries, the gene(s) of interest may be present at a low frequency. To circumvent this limitation, two main different strategies were implemented, such as (i) the development of cloning vectors that allow a positive selection or an easiest screening and (ii) the enrichment of a microbial community with a biological function of interest under appropriate conditions [31]–[32]. With respect to QS, functional metagenomics extended knowledge of the diversity of both the NAHL-producing and NAHL-degrading enzymes [9], [18]–[19], [33]–[34]. This approach also revealed biosynthesis of novel mimics which activate QS-response [35]. To our knowledge, a biased-environment, enriched in NAHL-degrading bacteria, was never used for discovering novel NAHLases by functional metagenomics.

In this work, γ-caprolactone (GCL), a biodegradable compound structurally related to NAHLs [26], was introduced in the rhizosphere of *Solanum tuberosum* to increase the percentage of NAHL-degrading bacteria in the plant environment. The GCL-induced bias on bacterial diversity was verified by a combination of *rrs*-DGGE and *rrs*-pyrosequencing. Then, genes involved in NAHL-degradation were searched among a library of 29,760 clones using a functional screening. A *qsdB*-encoded amidase was identified and characterized. This work is a first report of a NAHL-amidase that belongs to the amidase signature (AS) family, members of which are usually involved in the degradation of various xenobiotic compounds, such as nylon and linuron herbicide.

## Materials and Methods

### Plant Culture

One hundred plants of *S. tuberosum* var. Allians, which were recovered from cultures performed under axenic conditions, were placed into holes (3 cm space to each other) of batch lids. Each of the non-sterile batches (40×60×8 cm) contained 13 L of the nutritive solution Hydrobloom (Cellmax, UK) with nitrogen at 0.80 g/L, phosphore at 0.56 g/L and potassium at 1.48 g/L as major components. The solution was diluted from a concentrated stock solution (x250) with non-sterile water from the public water system. Planted batches were placed in the greenhouse (Comité Nord Plants de Pomme de Terre, Bretteville-du-Grand-Caux) under natural light at 10–15°C (night) and 25–30°C (day). Treatments (0.4 g/L) with γ-caprolactone (GCL; CAS # 695-06-7; Sigma-Aldrich) were performed at 1 and 28 days. This GCL concentration was selected because of its capacity to stimulate the growth of HAHL-degrading bacteria and its low toxicity to potato plant. A second batch was used as an untreated control. The two batches (untreated, GCL-treated) were simultaneously analyzed at 42-days. GCL concentration in batch and plant tissues was determined by HPLC-MS as previously described [36].

### Identification of Bacterial Isolates for NAHL-production and NAHL-degradation

At 42 days, one gram of roots (fresh weight) was suspended in 10 ml of sterile 10 mM MgSO_4_, diluted and spread onto TSA medium (AES) for counting members of the cultured bacterial community. Three samples were analyzed from each batch. From each of the six samples, thirty TSA-isolates were grown in 96-microwell plates and individually assayed for production of NAHLs with the *A. tumefaciens* biosensor NT1(pZNLR4) [37]. All the isolates were tested for their capacity to disrupt perception of the QS-signal hexanoylhomoserine lactone (C6HSL) using the biosensor *Chromobacterium violaceum* CVO26 [38].

### *rrs*-DGGE and *rrs-*pyrosequencing

PCR-amplicons of the *rrs* 5′-region between positions 341 and 534 (according to *E. coli* sequence) were submitted to DGGE analysis (Microbial Insights, Rockford, TN, USA) as previously described [39]. Sequence identification of selected bands was performed using the sequence match facility of the Ribosomal Database Project (http://rdp.cme.msu.edu/) according to NCBI taxonomy (http://www.ncbi.nlm.nih.gov/Taxonomy/).

Experimental procedures for *rrs*-amplification using 8F- and 357R-derived primers, 454-pyrosequencing (Roche/454 FLX Pyrosequencer at GATC biotech, Konstanz, Germany), and data analysis were previously described by Cirou et al. [36]. The obtained sequences were selected by their length (>150 nt) and their quality score (90% of nucleotides with a quality score>25) using the Greengenes website (http://greengenes.lbl.gov/cgi-bin/nph-index.cgi), and analyzed using the RDP's pyrosequencing pipeline at the Ribosomal Database Project Website (http://pyro.cme.msu.edu/). The reads were aligned with the Infernal Aligner software and complete linkage clustering method were performed to define OTU. Singleton OTUs were kept for the calculation of ecological indexes. CHAO1 and Shannon's Index were calculated at 0.03 and 0.05 distance.

### Quantification of *qsdA, qsdB and attM* Genes by qPCR

At 42 days, 10 L of nutritive solution from the untreated and GCL-treated batches were filtrated through a filter paper to eliminate large plant debris, then centrifuged at 17,600 g. Total genomic DNA was extracted using the DNesay Blood and Tissue Kit (Qiagen) according to the manufacturer’s protocol and quantified with a spectrophotometer (NanoDrop ND1000, Labtech, France). Relative abundance of the *qsdA, attM* and *qsdB* genes was estimated by quantitative PCR (qPCR) in 96-well plates using a LightCycler 480 (Roche). Primers were designed for conserved region of the genes *qsdA* (5′-ACGAGCATGTCTTCGTTCTG and 5′-GGATCGACGA TCGTGCTGAT), *attM* (5′-TGACATCGGCCGGATCGAAA and 5′-ACGGCGGC AACGCGATTGAA), and *qsdB* (5′-GAGTGCCCAGGAACTTCACG and 5′-CCTTGAT CAGGAAGGGCACG). Primer couples generated fragments of ca. 150 bp in length. Calibration curves of *qsdA*, *attM*, and *qsdB* were defined with genomic DNA of *Rhodococcus erythropolis* R138, *Agrobacterium tumefaciens* C58 and *E. coli* DH5α harboring the p90H6 fosmid, respectively. Composition of the PCR mix for each sample was as follows: 5 µL of SYBER Green I Master Mix (Roche), reverse primer (0.5 µM), forward primer (0.5 µM) and 1 µL of DNA sample at 8 ng/µl. Calculation of the relative abundance of the investigated genes *attM*, *qsdA*, and *qsdB* in batches was normalized according level of *attM* gene in untreated batch and took into account the variation of DNA concentration in batches.

### Construction and Screening of the Metagenomic Library

The metagenomic library of the microbial community collected at 42 days from the GCL-treated batch was obtained as follows. DNA was partially digested with Sau3A. The resulting fragments (40–50 kbp) were cloned into the fosmid EpiFos harboring a chloramphenicol-resistance gene, and transferred into *E.coli* DH10B (LibraGen, Toulouse, France). A total of 29,760 clones constituted the metagenomic library which was distributed into 310 microwell plates. Each clone was individually cultivated in 96-well plates and tested for inactivation of the C6HSL signal (12.5 µM), according the previously described procedures [9].

### Sequencing and Phylogenetic Analyses of the NAHL-degrading Fosmid p90H6

Complete sequence of the p90H6 fosmid (46 kbp) harboring a 39 kbp insert was achieved by Sanger sequencing. To this end, a library of 2.0–2.5 kbp fragments was constructed, cloned and sequenced by GATC Biotech (Mulhouse, France). Assembling (7x in average coverage) was performed using DNASTAR Lasergene, and appropriate primers were designed for gap-closure. DNA map was generated by Geneious and *orf* determination by Bioedit softwares. For each of the putative proteins encoded by the p90H6 insert, 50 homologous sequences were retrieved from GenBank (http://www.ncbi.nlm.nih.gov) after identification by BLASTp. Multiple sequence alignments were carried out with ClustalW. The evolutionary history was inferred using the Maximum Likelihood method based on the JTT matrix-based model [40]. The percentage of replicate trees in which the associated taxa clustered together in the bootstrap test (500 replicates) was calculated [41]. Initial tree(s) for the heuristic search were obtained automatically as follows. When the number of common sites was <100 or less than one fourth of the total number of sites, the maximum parsimony method was used; otherwise BIONJ method with MCL distance matrix was used. Evolutionary analyses were conducted inMEGA5 [42].

### NAHL-degradation Assays by Entire Cells and Cell-free Extract

The pMTHindIII and pMTXhoI plasmids were pME600 derivatives harbouring a 4.5 kbp and 2.3 kbp *qsdB*-insert, respectively. Overnight cultures of *E.coli* strain DH5α harboring the p90H6, pME6000 empty vector or the two plasmids pMTHindIII and pMTXhoI were adjusted to OD_600 nm_ 2.0. The resulting suspensions were incubated at 30°C with C6HSL at 25 µM, OC8HSL and C8HSL at 400 nM in LBm medium. Assays were performed up to 48 h, and residual concentration of NAHL was determined using the NAHL biosensors *A. tumefaciens* biosensor NT1(pZNLR4) and *C. violaceum* CVO26.

NAHL-degradation assay was also performed using cell-free extract of *E. coli* harboring the pME6000 and pMTXhoI plasmids. Detection of residual C6HSL was done by HPLC-MS. Overnight cultures of the *E.coli* strains were centrifuged, washed twice in KPBS buffer (10 mM Na_2_HPO_4_ 1.76 mM KH_2_PO_4_ pH 6.8), and sonicated in the presence of a protease inhibitor cocktail (Sigma P8465). Cell debris were removed by centrifugation (20 min, 4°C, 14,000 g), and the resulting crude cell extract was filtered through polyethersulfone column (10 kDa Vivaspin500). In NAHL-degradation assay, the reaction mixture contained cell free extract at a final concentration of 10 mg/mL and C6HSL at 50 µM in a final volume of 1 ml of KPBS buffer. Assays were incubated at 30°C up to 24 h, and filtered through polyethersulfone column (10 kDa Vivaspin500). Thirty to 50 µL were analyzed by HPLC (Waters Alliance 2690) conjugated with LC-MS/MS (Waters ZQ Mass Spectrometer with single quadrupole system and electrospray ionization). To detect C6HSL, a Gemini C18 5 µm (2.0×150 mm) column was eluted with an isocratic mixture (80/20) of H2O/HCOOH (0.1%) and CH3CN/HCOOH (0.1%). Quantification was performed according a calibration curve generated with pure C6HSL.

### Soft Rot Tuber Assays

Cultures of *Pectobacterium atrosepticum* CFBP6276 and its derivatives harboring the pME6000 and pMTXhoI plasmids were washed twice in NaCl 0.8%. Fourteen tubers of *S. tuberosum* var. Allians were inoculated with 10^7^ CFU of the plant pathogen, and incubated at 25°C. Five days after infection, the tubers were cut in the middle, and observed. The aggressiveness of the strains was categorized into four classes of diameters of the maceration zone: less than 1 mm, between 1 and 2 mm, between 2 and 5 mm, more than 5 mm. The Kruskal–Wallis test (α = 0.05) allowed the statistical analysis of the maceration categories.

### Construction and Purification of the His-tag Derivatives of QsdB, LibA and NylA

The *qsdB*, *libA* (G3K3F8 UniProt KB) and *nylA* (P13398 UniProt KB) genes were synthezised by Eurofins (Germany). Codon usage was optimized for heterologous expression in *E. coli*. Appropriate restriction sites and six terminal His-codons were incorporated for cloning the genes in the pET11d expression vector. Mutations in the catalytic triad K-S-S of QsdB were constructed using the pET11d-*qsdB* vector as a template and the QuikChange® II XL site-directed mutagenesis kit (Stratagene). The synthetic forward primers 5′-TTCCTGATCGCAGACCTGGTT (the altered nucleotides underlined), 5′-TCGGGTGGTGCCTCTGGAGGG, and 5′-GGCGGTGGTGCTATCC GCATT, and their appropriate reverse primers were designed for the mutations K70A, S147A, and S171A, respectively. All constructed alleles were verified by DNA-sequencing (GATC Biotech, Mulhouse, France).

QsdB and LibA expression plasmids were transformed into *E.coli* Rosetta pLysS (Novagen), and NylA expression plasmid in *E.coli* C41 (Lucigen). The cells were grown in 2YT media at 37°C to OD_600 nm_ 0.6; the temperature was reduced to 28°C, and expression was induced with 0,5 mM IPTG during 5 h. The cells were harvested and stored at −20°C. The pellet was suspended in buffer A (50 mMTris–Cl buffer, pH 8, 20 mM imidazole, 10% glycerol and 500 mM NaCl) and disrupted by sonication. The lysate was cleared by centrifugation at 20,000 g for 30 min at 4°C. The supernatant was filtered through a 0.22 µm membrane (Millipore Stericup) and loaded on a 5 ml Ni-NTA agarose column (GE Healthcare). After washing with 10% of buffer B (50 mMTris–Cl pH 8, 300 mM imidazole, 10% glycerol and 500 mM NaCl), elution was performed with 100% of buffer B. Fractions containing the proteins were pooled, and a buffer exchange was performed on a Superdex 200 10/300 (GE Healthcare) or by dialysis (SlideA Lyzer, Pierce) during 4 hours in Tris 50 mMTris–Cl pH 8, 150 mM NaCl and 10% glycerol. Fractions were analyzed by SDS-PAGE, and those containing the proteins were pooled, concentrated using Vivaspin-10 kDa (Sartorius) and stored at −20°C.

### HPLC-MS Identification of Homoserine Lactone Released by the Amidase QsdB

Purified QsdB were incubated at 0.1 mg/mL with 12.5 µM of C6HSL for 24 hours, then 200 µL of the assay was filtered through polyethersulfone column (10 kDa Vivaspin500). The eluate was diluted 1/5 in acetonitrile (CH3CN), and finally, 5 µL were in the HLC column for detection of homoserine lactone, as an amidase product of C6HSL. To detect HSL molecule, a SeQuant Zic-pHilic 5 µM (150 mm×4.6 mm) column was eluted by isocratic mixture (80/20) of H2O/HCOOH (0.1%) and CH3CN/HCOOH (0.1%). Five µL of standard HSL in buffer elution 1% and acetonitrile/water at a ratio 80/20 at different concentrations were also injected through the column to generate a calibration curve.

## Results

### High Proportion of NAHL-degrading Bacteria in GCL-treated Environments

**Figure 1:**
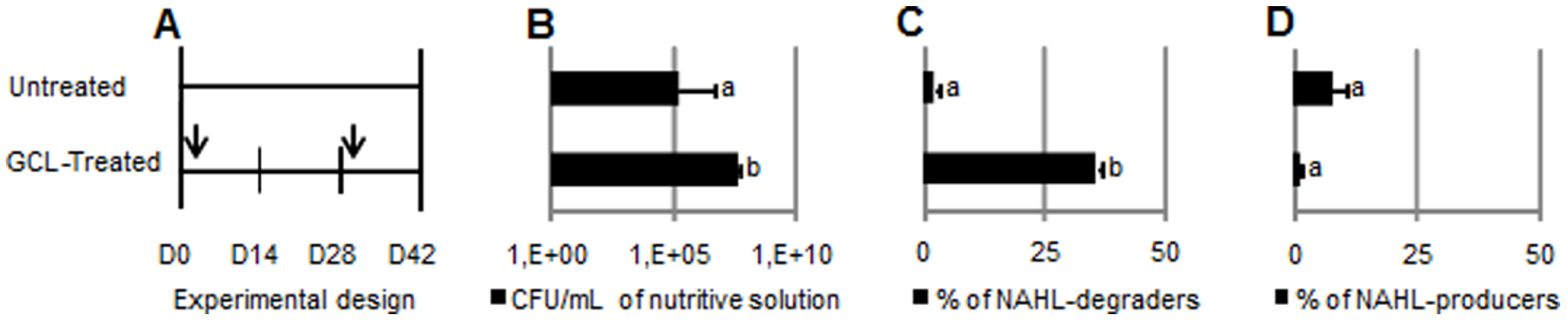
NAHL-degrading community in GCL-treated plant cultures. (A) Potato plants were cultivated under untreated and GCL-treated conditions during 42 days; vertical arrows indicate the two applications of GCL at 0.4g/L. At 42-day, total cultured bacteria (B), and percentage of NAHL-degraders (C) and NAHL-producers (D) were enumerated and calculated under both the GCL-treated and untreated conditions. The mean of three replicates are shown. Statistically different values (Mann & Whitney, α = 0.05) are noted by different letters.

GCL (0.4 g/L) was introduced twice (1-day and 28-day) in the rhizosphere of *Solanum tuberosum* var. Allians grown under hydroponic conditions (Fig. 1A). At 42-day, cultured bacteria (CFU/g of fresh root) were significantly more abundant in the GCL-treated batch than in the untreated one (Fig. 1B). Among cultured bacteria, the proportion of C6HSL-degrading bacteria reached 3% in control batch, and 36% in the treated batch (Fig. 1C). In contrast, no significant modification of the percentage of NAHL-producing bacteria was observed in the different batches (Fig. 1D). Partial *rrs*-sequencing showed that all isolated NAHL-degrading bacteria belong to the *Rhodococcus* genus. Using high performance liquid chromatography-mass spectrometry (HPLC-MS), GCL concentrations were measured in nutritive solutions and plant tissues of untreated and GCL-treated cultures at 42-day. While GCL was introduced at 0.8 g/L (2×0.4 g/L), it was detected at 3.2 10^−4^ g/L in the nutritive solution and at 4.9 10^−4^ mg/g of fresh weight in plant tissues recovered from treated batch at 42-day. GCL was undetectable in the untreated samples. However, over the same period, GCL concentration remained stable in a sterile nutritive solution.

### Drastic Reshaping of Bacterial Diversity Upon GCL-treatment

**Figure 2:**
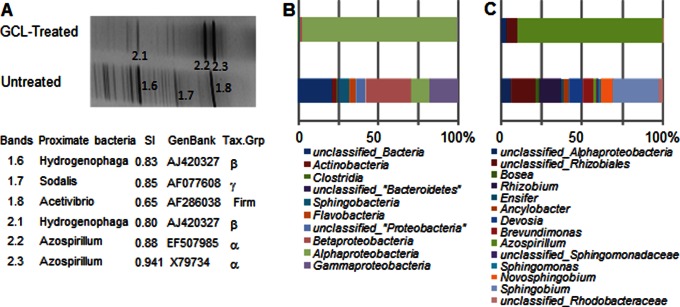
GCL-induced changes in the bacterial *rrs*-diversity. At 42-day, *rrs*-diversity was evaluated by DGGE (A) and pyrosequencing (B, C). In A, for each of the numbered bands, one of the closest *rrs* sequences was indicated and characterized by GenBank number, the name and taxonomical position of the bacteria of origin (Firm, Firmicutes; α, α-Proteobacteria; β, β-Proteobacteria; γ, γ-Proteobacteria), and the similarity index (SI) calculated at Ribosomal Database Project (http://rdp.cme.msu.edu/). The *rrs*-pyrosequencing data were analyzed at the class level (B) and, within the class of alphaproteobacteria, at the genus level (C).

GCL-mediated modifications of bacterial diversity in plant environment were explored by *rrs*-DGGE and *rrs*-pyrosequencing. After 42 days, total DNA was extracted from the bacterial cells collected from the nutritive solution and used as a template for *rrs*-amplifications. DGGE profiles revealed a drastic remodeling of bacterial populations after GCL treatments (Fig. 2A). Under GCL-treated conditions, major bands identified by sequencing were generated from members of the *Azospirillum* genus, while in untreated control they originated from members of the genera *Hydrogenophaga* and *Acetivibrio*.

The *rrs*-pyrosequencing (NCBI accession number PRJNA196890) gave a deeper view of bacterial community and confirmed the high abundance of *Azospirillum*-related bacteria in the GCL-treated samples. All 86,960 usable sequences (36,847 and 50,113 associated to the untreated and GCL-treated samples, respectively) were analyzed by hierarchical clustering to define clusters at 97% and 95% sequence identity. These served as Operational Taxonomy Units (OTUs) for calculation of Chao1 and Shannon indexes. In the untreated samples, cluster number reached 834 (610 with 95% cut-off), whereas it was only 271 (188 with 95% cut-off) in GCL-treated samples. Using the 97% cut-off, the Chao1 and Shannon values were higher in the untreated sample (1204 and 4.03, respectively) than in treated samples (397 and 1.94). Similar data were obtained with the 95% cut-off: the Chao1 and Shannon values were 824 and 4.00 under untreated condition and 309 and 1.29 under GCL-treatment. These data indicate that GCL-treatment strongly stimulated the growth of a limited number of OTUs, and hence reduced the diversity of the resident community. Diversity analysis of the GCL-induced bias was refined by identifying and comparing *rrs*-sequences at the class and genus levels using tools available from the Ribosomal Database Project Website. While α-proteobacterial *rrs*-sequences dominated in GCL-treated samples (more than 95%), those identified in the untreated samples belonged mostly to β-proteobacteria (26%), unclassified bacteria (21%) and γ-proteobacteria (18%) (Fig. 2B). As mentioned above, *Azospirillum* appeared as the dominant genus that represented up to 92% of the identified α-proteobacteria in treated samples (Fig. 2C). In contrast, the α-proteobacterial diversity of untreated batch samples was larger, with more than 10 genera of which emerged *Sphingobium* (30%) and *Rhizobium* (15%).

### Identification of a Metagenomic Fosmid Encoding NAHLase Activity

Bacterial DNA was extracted from the GCL-treated batch at 42-day, and used to construct a metagenomic library of 29 760 fosmids, each of which exhibited a DNA insert of 40 to 50 kbp. Hence, the metagenomic library represented up 1.5 Gbp of environmental DNA. All metagenomic clones were individually tested for their capacity to disrupt QS using hexanoyl-homoserine lactone (C6HSL) as a prototypic signal and *Chromobacterium violaceum* CVO26 as a bacterial biosensor. A single fosmid, p90H6, conferring NAHL-degradation ability was identified and sequenced.

The fosmid p90H6 contains an insert of 39,632 bp encoding 34 complete ORFs (NCBI accession number JQ292794). Protein sequence comparison of all the deduced ORFs was performed using BLAST searches upon available sequences in GenBank database. This analysis revealed that out of 34 ORFs, 31 showed highest similarity with known or predicted proteins from bacteria belonging to the phylum of Proteobacteria. Furthermore, a phylogenetic analysis of 50 homologous proteins was carried out for each of the 34 ORFs allowing the identification of the closest orthologs which are showed in the Table 1. Phylogenetical approach confirmed the relationship between DNA insert of p90H6 and the class of Proteobacteria, and especially γ-proteobacteria. Two remarkable gene clusters were identified. The first one from *orf12* to *orf18* could be involved in metabolism of acetophenone-related compounds; the second one from *orf25* to *orf34* in the catabolism of the plant hormone indole-3-acetic acid. Another remarkable feature was the similarity of *orf1*, which is located immediately downstream the *lacZ*-promoter of the fosmid, with amidase or putative amidase genes. This *orf*, studied in this work, was named *qsdB* for quorum-sensing degradation.

**Table 1:** ORFs of the p90H6 DNA-insert.

ORF n°	Peptide length	Hypothetical function(automatic annotation)	Accession n°	Organism	Phylum
1	479	Putative amidase	YP_372308.1	*Burkholderia cenocepacia* J2315	β-Proteobacteria
2	117	Inner-membrane translocator	YP_001264838.1	*Sphingomonas wittichii* RW1	α-Proteobacteria
3	719	Hypothetical protein	YP_001264839.1	*Sphingomonas wittichii* RW1	α-Proteobacteria
4	438	Hypothetical protein	NP_901586.1	*Chromobacterium violaceum* ATCC 35937	β-Proteobacteria
5	424	Cobalamin biosynthesis CobW-like domain-containing protein	YP_002798347.1	*Azotobacter vinelandii* DJ	γ-Proteobacteria
6	388	Acetyl-CoA acetytransferase	YP_524523.1	*Rhodoferax ferriducens* T118	γ-Proteobacteria
7	509	AMP-dependent synthetase andligase CoA	YP_524524.1	*Rhodoferax ferriducens* T118	γ-Proteobacteria
8	240	Succinyl-CoA-3-ketoacid-CoA transferase subunit A	YP_001854087.1	*Kocuria rhizophila* DC2201	Actinobacteria
9	211	3-oxoacid CoA-transferasesubunit B	YP_001235372.1	*Acidiphilum cryptum* JF-5	α-Proteobacteria
10	674	σ54-dependent activator protein	YP_002798346.1	*Azotobacter vinelandii* DJ	γ-Proteobacteria
11	60	No match	-	*-*	-
12	645	Acetophenone carboxylase	YP_002798345.1	*Azotobacter vinelandii* DJ	γ-Proteobacteria
13	132	Acetophenone carboxylasesubunit γ	YP_002798344.1	*Azotobacter vinelandii* DJ	γ-Proteobacteria
14	436	Acetophenone carboxylase	YP_002798343.1	*Azotobacter vinelandii* DJ	γ-Proteobacteria
15	412	Acetophenone carboxylase	YP_002798343.1	*Azotobacter vinelandii* DJ	γ-Proteobacteria
16	620	Hydantoinase B/oxoprolinase	YP_001416247.1	*Xanthobacter autotrophicus* Py2	α-Proteobacteria
17	118	Acetophenone carboxylase	YP_002798342.1	*Azotobacter vinelandii* DJ	γ-Proteobacteria
18	285	Hypothetical protein	YP_002798341.1	*Azotobacter vinelandii* DJ	γ-Proteobacteria
19	167	Zinc/iron permease	ZP_08503466.1	*Methyloversatilis universalis*FAM5	β-Proteobacteria
20	183	No match	-	*-*	-
21	338	Hypothetical protein	YP_159683.1	*Aromatoleum aromaticum* EbN1	β-Proteobacteria
22	794	RND superfamily exporter	YP_159684.1	*Aromatoleum aromaticum* EbN1	β-Proteobacteria
23	516	Transcriptional regulator LysR	YP_159685.1	*Aromatoleum aromaticum* EbN1	β-Proteobacteria
24	436	Hypothetical protein	YP_0001020138.1	*Methylibium petroleiphilum* PM1	β-Proteobacteria
25	321	Amidase	YP_003451629.1	*Azospirillum* sp. B510	α-Proteobacteria
26	387	Hypothetical protein	YP_004590677.1	*Enterobacter aerogenes* KCTC 2190	γ-Proteobacteria
27	119	Hypothetical protein	EGC94464.1	*Escherichia fergusonii* ECD227	γ-Proteobacteria
28	173	Hypothetical protein	ABY62766.1	*Pseudomonas putida*	γ-Proteobacteria
29	421	Ring hydroxylating subunit alpha (IacC)	YP_002381972.1	*Escherichia fergusonii*ATCC 35469	γ-Proteobacteria
30	158	Aromatic ring hydroxylating dioxygenase beta subunit (IacD)	ABY62760.1	*Pseudomonas putida*	γ-Proteobacteria
31	240	Short-chain dehydrogenase/reductase SDR (IacE)	YP_04381977.1	*Pseudomonas mendocina* NK-01	γ-Proteobacteria
32	320	Ferredoxin (IacF)	YP_04381976.1	*Pseudomonas mendocina* NK-01	γ-Proteobacteria
33	166	Flavin reductase-like protein (IacG)	YP_346445.1	*Pseudomoans fluorescens* Pf0-1	γ-Proteobacteria
34	134	Putative tautomerase	YP_004415698.1	*Pusillimonas* sp. T7-7	β-Proteobacteria

### Relative Abundance of *qsdB* in GCL-treated Bacterial Community

**Figure 3:**
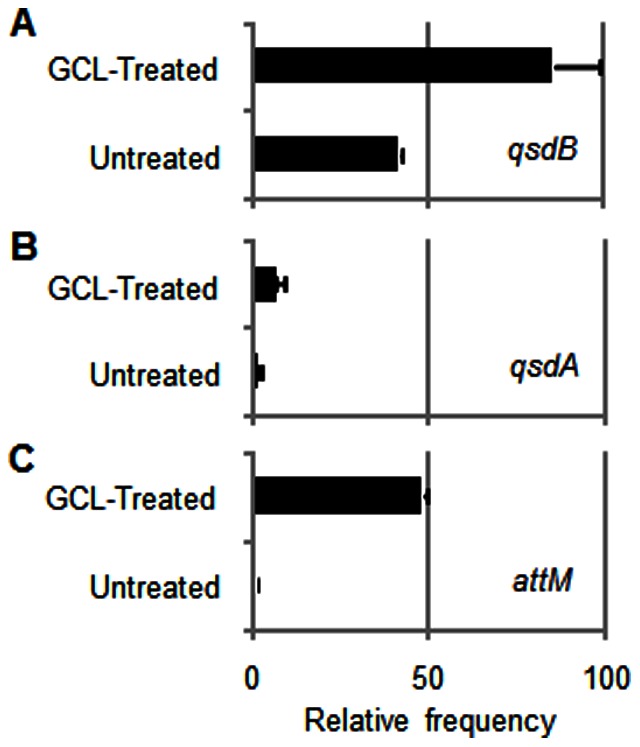
Relative abundance of NAHLase-encoding genes in GCL-treated and untreated plant cultures. Relative abundance of the *qsdB* (A), *qsdA* (B) and *attM* (C) genes in GCL-treated and untreated batches at 42-day was measured by qPCR. The *attM* intensity under the untreated condition (C) was used as a normalized reference (arbitrary value = 1) for calculation of the relative abundance of all genes.

In parallel to the enzymatic characterization of QsdB, the relative abundance of genes encoding NAHL-degrading enzymes was compared by qPCR in the DNA extracted from the GCL-treated and untreated rhizosphere. In addition to *qsdB*, we quantified *qsdA* from *Rhodococcus erythropolis* [12] the proportion of which increased after GCL-treatment [36], and *attM* which is present in *Agrobacterium* [6] and related α-proteobacteria. The lowest level of *attM* in the untreated condition was arbitrary chosen as a reference level. The relative abundance of *qsdA* and more notably that of *qsdB* and *attM* were higher in GCL-treated batch than in untreated batch (Fig. 3). However, in the GCL-treated condition, the relative abundance of *qsdB* was higher than that of *attM* and *qsdA*.

### Characterization of *qsdB* and Encoded NAHL-amidase Activity

**Figure 4:**
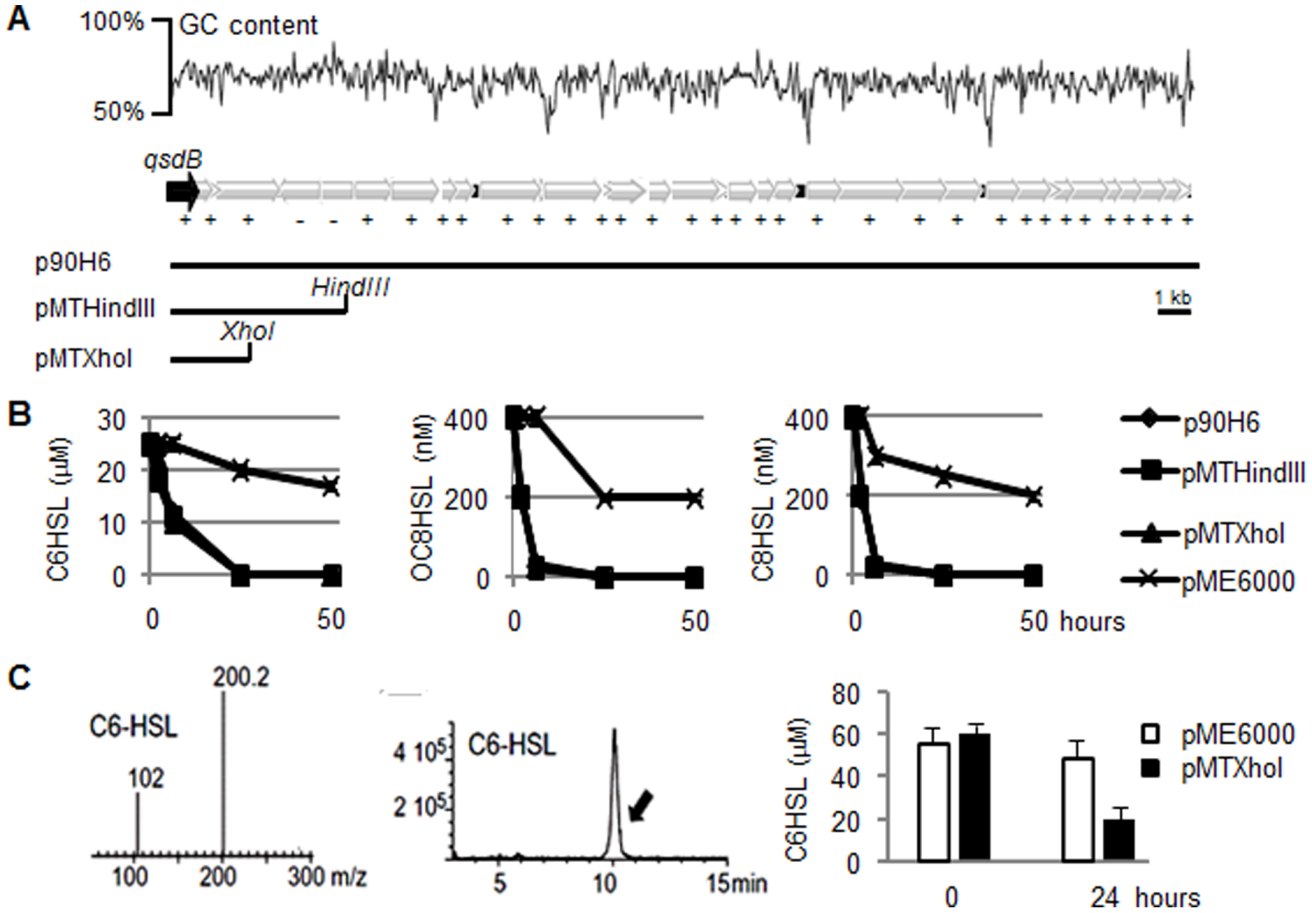
Physical map of the fosmid p90H6 and sub-cloning of *qsdB* coding for NAHLase activity. In A, GC content (%) and orientation (+/−) of the 34 putative *orfs* of the fosmid p90H6, and physical map of the pME6000-derivatives pMTHindIII and pMTXhoI barboring *qsdB* (*orf1*). In B, residual level of C6HSL, OC8HSL or C8HSL measured in the presence of *E. coli* strain DH5α harboring the empty vector pME6000, the fosmid p90H6, and the constructed pMTHindIII and pMTXhoI (symbols of the three formers are superimposed in the graphs).Three replicates were done. In C, C6HSL analysis by HPLC/MS: mass, retention time, and quantification of C6HSL before (t = 0) and 24 hours after incubation in the presence of cell-free extracts of *E. coli* strains DH5α(pME6000) and DH5α (pMTXhoI).

The GC content of the metagenomic DNA-sequence reached a mean value of 67.2%, and appeared to be roughly stable throughout the whole insert, though some GC% differences could be seen in between some ORFs (Fig. 4A). Two pME6000 plasmid derivatives, pMTHindIII and pMTXhoI harboring *qsdB* on 4.5 kbp and 2.3 kbp inserts, respectively, were constructed (Fig. 4A). In addition to *qsdB*, the pMTXhoI smallest insert contains an additional *orf* encoding a putative inner-membrane translocator (117 amino acids) with no expected hydrolytic properties. *E. coli* cells harbouring p90H6, pMTHindIII or pMTXhoI were able to inactivate over 95% of the introduced NAHLs, which were C6HSL, octanoylhomoserine lactone (C8HSL), and 3-oxo-octanoylhomoserine lactone (OC8HSL), in less than 24 hours (Fig. 4B). A HPLC-MS analysis confirmed the disappearance of the QS-signal C6HSL in the presence of cell-free extract from the *E. coli* strain harboring the pMTXhoI plasmid (Fig. 4C).

**Figure 5:**
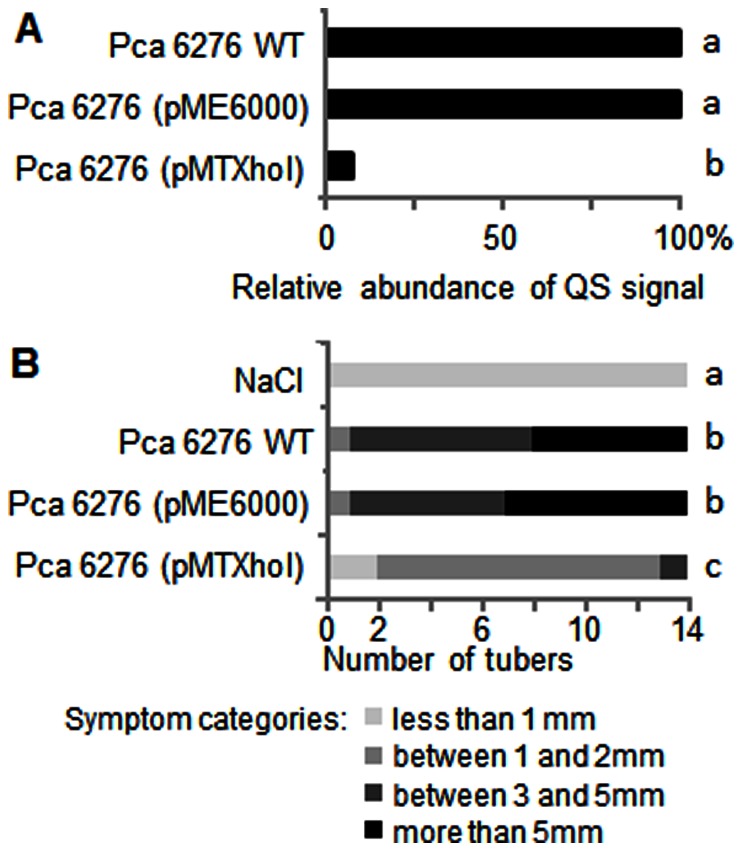
QsdB-mediated quorum-quenching in *P.* *carotovorum.* In A, relative abundance of NAHLs (OC8HSL as the main QS-signal) in wild-type *P. carotovorum* CFBP6276 (Pca6276), and *P.carotovorum* harboring the empty vector pME6000 and the pMTXhoI plasmid expressing QsdB. In B, symptoms on potato tubers (n = 14) inoculated with Pca6276, Pca6276 (pME6000), Pca6276 (pMTXhoI) or NaCl 0.8% as a negative control were classified according to their importance (see material and methods) and compared using Kruskal-Wallis test (P<0.05). Different letters indicate statistical differences between the compared plant pathogens.

**Figure 6:**
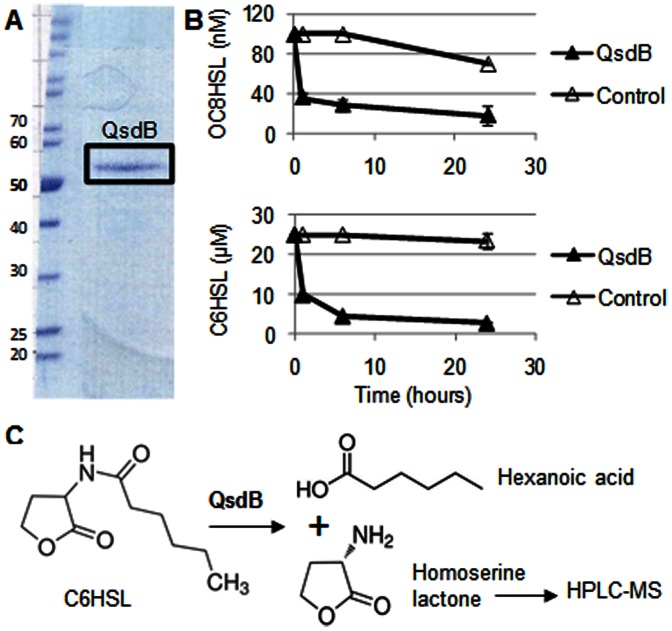
Purification and amidase activity of QsdB. In A, SDS-PAGE analysis of the S-200 fraction containing the purified QsdB. In B, degradation kinetics of OC8HSL and C6HSL in the presence of QsdB at 0.1 mg/ml; the protein-free buffer was used as a control. Three replicates were done. In C, Amidase activity of QsdB toward C6HSL releases hexanoic acid and homoserine lactone which was characterized and quantified by HLPC/MS as described in the material and method section.

To evaluate the quorum-quenching capacity of the pMTXhoI plasmid, this later was introduced into the plant pathogen *P. atrosepticum* CFBP6276, in which expression of virulence factors is controlled by QS with OC8HSL as a major signal [43]. The constructed *P. atrosepticum* (pMTXhoI) exhibited a ten fold decrease of the NAHL level (Fig. 5A) and a reduced level of symptoms on potato tubers (Fig. 5B) as compared to the control strain *P. atrosepticum* (pME6000). Finally, the QsdB enzyme was purified using a His-tag procedure (Fig. 6A), verified for its capacity to inactivate QS-signals (Fig. 6B), and tested for amidase activity using HPLC-MS identification and quantification of homoserine lactone as a reaction product (Fig. 6C). After 24-h incubation in the presence of QsdB, 100% of the introduced C6HSL (13 µM) were converted into the amidase product homoserine lactone, the concentration of which reached 14±1 µM.

### QsdB Belongs to the Amidase Signature (AS) Family and Requires the Catalytic Triad K-S-S for NAHLase Activity

**Figure 7:**
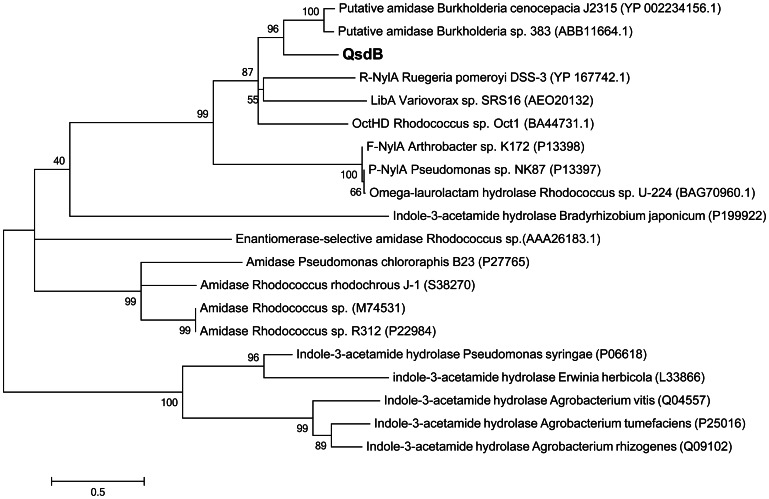
QsdB within the amidase signature (AS) family. Phylogenetic relationship between twenty AS-family members including the metagenomic NAHL-amidase QsdB was inferred using the Maximum Likelihood method based on the JTT matrix-based model. Percentage of the replicate trees in which the associated taxa clustered together in the bootstrap test (500 replicates) is shown next to the branches. The tree is drawn to scale, with branch lengths measured in the number of substitutions per site.

BLAST analysis highlighted the AS family enzyme OctHD (47% identity with QsdB) among the 50 closest relatives of QsdB. A refined phylogenetic analysis of QsdB confirmed its position within the AS family (Fig. 7). QsdB belongs to a sub-cluster which encompasses the NylA proteins from *Ruegeria*, *Arthrobacter* and *Pseudomonas*, the ω-laurolactam and ω-octalactam hydrolases from *Rhodococcus*, the linuron-degrading enzyme LibA of *Variovorax*, and two predicted amidases in *Burkholderia cenocepacia* J2315 and *Burkholderia* sp. 383. To evaluate whether the NAHLase activity is conserved within the AS family, the proteins LibA and NylA were purified using the same His-tag approach as described for QsdB. The purified LibA and NylA did not inactivate NAHLs suggesting a probable specialization of these enzymes towards their xenobiotic substrates.

**Figure 8:**
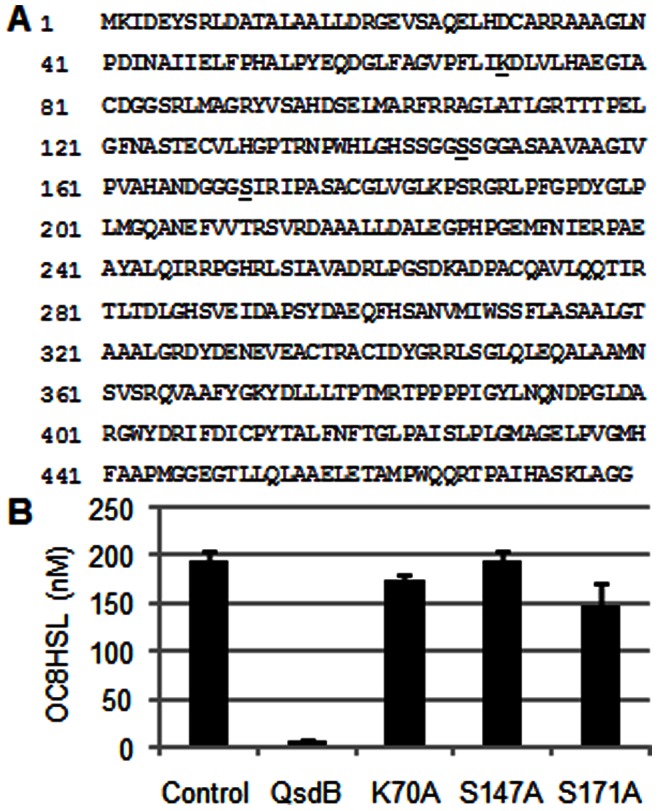
The AS-family catalytic triad K-S-S is required for NAHLase activity of QsdB. In A, the amino acids K70, S147 and S171 of the AS-family catalytic triad are underlined in the QsdB sequence. In B, concentration of residual OC8HSL after 24 hr-incubations in the presence of the wild type protein QsdB and its constructed derivatives K70A, S147A and S171A, all at 0.1 mg/ml; the protein-free reaction buffer was used as a control.

Three residues, collectively named the catalytic triad K-S-S, have been described as being involved in the amidase activity of several AS family members [44]–[46]. A sequence alignment revealed that these amino-acid residues were also conserved in QsdB at positions Lys70, Ser147, and Ser171 (Fig. 8A). To evaluate their implication in NAHLase activity, three mutants of QsdB affected in each of the K-S-S residues were constructed, purified and tested against NAHLs (Fig. 8B). All constructed protein mutants lost their NAHLase activity as compared to the wild type QsdB, a result that demonstrates the involvement of the AS-family triad K-S-S in the degradation of the QS signals.

## Discussion

The novel metagenomic NAHL-amidase QsdB identified in this study is distantly related to the previously identified NAHLases and belongs to the AS family that comprises most than 200 proteins from diverse origins including Bacteria, Archaea and Eukarya [44]. The amidase activity of AS enzymes requires the K-S-S triad [44]–[45], which is also involved in the NAHLase activity of QsdB. Despite their phylogenetic proximity and common ability to hydrolyse amide bonds, individual representatives of the AS family show very distinctive substrate-specificities [45]. It has been suggested that this is due to the involvement of residues outside the catalytic triad K-S-S in substrate binding [44]. Remarkably, QsdB belongs to an AS sub-cluster, which encompasses the bacterial amidases NylA, OctHD and LibA cleaving xenobiotic compounds such as nylon and the herbicide linuron [47]–[49]. To our knowledge, QsdB is the first enzyme of the NylA-OctHD-QsdB-LibA sub-cluster to be involved in the degradation of natural compounds, i.e. NAHLs. One can hypothesized that QsdB-related NAHLases would be ancestors from which have evolved the enzymes cleaving structurally-related xenobiotics, such as linuron and nylon. The pre-existing skeleton of AS-family NAHLase would therefore explain, in part, why nylon- and linuron-degrading enzymes have rapidly emerged in microbial communities even though such compounds have been only recently introduced in the soil as a consequence of industrial and agricultural activities [50]–[51]. Such evolved genes encoding for substrate-specialized enzymes could be thereafter propagated by horizontal transfer [50].

QsdB-encoding gene belongs to a large environmental-DNA insert (39 kpb) of which most of the ORFs (31/34) were phylogenetically associated with *Proteobacteria.* In addition to *qsdB*, this environmental DNA harbored two remarkable regions, from *orf12* to *orf18* and from *orf25* to *orf34*, which could be involved in the metabolism of acetophenone-related compounds and indole-3-acetic acid. Nine of the 10 genes which are known to be involved in the catabolism of indole-3-acetic acid [52] are present in the metagenomic fosmid. Their syntheny designated γ-proteobacteria as the closer bacteria. The presence of genes encoding for auxin catabolism strongly suggests that the trapped environmental DNA could belong to a plant-associated bacterium. This feature is in line with the rhizospheric origin of the analyzed community. Noticeably, this metagenomic DNA did not show robust similarities with the known genomes of *Azospirillum* (α-proteobacteria) which was the most abundant genus in the analyzed environment, neither with *Rhodococcus* (Actinobacteria) which was the dominant genus among the isolated NAHL-degrading bacteria. Previous studies showed that, among bacterial isolates recovered from GCL-treated rhizosphere, the NAHL-degrading *Rhodoccocus* grow on GCL as a sole carbon source [53], while *Azospirillum* assimilate GCL, but can not inactivate NAHLs [26]–[27]. The qPCR analyses in metagenomic DNA confirmed that *qsdB* was most abundant than the genes *qsdA* which belongs to *Rhodococcus* and *attM* which is associated to *Agrobacterium* and related α-proteobacteria. However, the frequency of trapped NAHLase-expressing fosmid among the constructed library is quite similar to that observed in previous studies [9], [33]. The apparent low frequency of positive NAHL-degrading clones may be explained by the combined constraints of large size of the inserts (50 kbp) and necessity to drive the gene expression from the promoter located at the cloning site of the vector in an *E. coli* host (see reviews about functional metagenomics in [32], [54]).

Finally, this study increases our knowledge on the dynamics of bacterial community upon hydroponic culture of potato plants in green-house. While the previous 454-pyrosequencing analyses of *rrs*-amplicons focused on the early stages (0 to 4 weeks) of plant growth culture [36], this work focused on a later stage at 7 weeks. In the two analyzed conditions (with or without GCL-treatment), the α-proteobacterium *Azospirillum* dominated the bacterial 7-week community, while *Acidovorax* and *Flavobacterium* were the most abundant genera in the earliest communities [36]. This work highlights the remarkable capacity of the *Azospirillum* genus to take advantage of the simultaneous constraints that are plant and aquatic environments, as suggested by comparative genomics [55]. This analysis should be repeated in an independent experiment for robustly deciphering the community dynamics.

In a conclusion, this work highlights the power of metagenomics to access unknown functional diversity, and reveals a common phylogenetical origin between AS-family amidases involved in the degradation of cell-to-cell signals and xenobiotics.
